# Editorial: Understanding and treating rare nasal and paranasal sinus cancers

**DOI:** 10.3389/fonc.2025.1573770

**Published:** 2025-03-03

**Authors:** Maria Grazia Ghi, Paolo Bossi, Pierluigi Bonomo, Piero Nicolai

**Affiliations:** ^1^ Oncology Unit 2, Veneto Institute of Oncology (IRCCS), Padua, Italy; ^2^ Medical Oncology and Hematology Unit, Humanitas Research Hospital, Rozzano, Milan, Italy; ^3^ Department of Radiation Oncology, Careggi University Hospital, Florence, Italy; ^4^ Otorhinolaryngology and Head and Neck Unit, Department of Neurosciences, Azienda Ospedale Università di Padova, Padua, Italy

**Keywords:** sinonasal cancers, rare cancers, paranasal sinus tumors, nasal sinus tumors, treatment

This Research Topic focuses on rare nasal and paranasal sinus cancers. The annual incidence of these cancers is approximately 0,5-1 case per 100,000 population, representing less than 5% of all head and neck cancers (1, 2). They vary widely in histological type, aetiology, biology and prognosis. Due to their low prevalence, standard treatment strategies are often lacking, in part because they are typically excluded from clinical trials for head and neck cancers. Surgery represents the mainstay of treatment, but due to the frequent involvement of critical structures such as the orbit, nasal pyramid, internal carotid artery and brain, it often leads to functional and aesthetic sequelae that can severely impact quality of life.

The aim of this Research Topic was to collect articles on these rare and complex cancers, with the goal of refining the understanding of their biological and clinical profile.

Three original research articles and three case reports were published in this Research Topic.

Smaadahl et al. contributed by reporting a retrospective study of treatment modalities and associated morbidity in 156 patients with sinonasal malignancies of various histologies. Surgery was the primary treatment in approximately two-thirds of the patients, and adjuvant radiotherapy (with or without concurrent chemotherapy) was found to follow surgery in several cases. The authors found a negative association of some factors, mainly histology and skull base involvement, with local control and survival outcomes. In line with other reports (3), patients with mucosal melanoma had the worst prognosis, as well as non-surgically treated patients, reflecting the poor outcome of unresectable and inoperable patients.

Hoshi et al. presented a retrospective series of 11 patients treated with systemic therapy for incurable recurrent and/or metastatic (R/M) olfactory neuroblastoma (ONB). ONB accounts for approximately 2-3% of all sinonasal tumors. It is usually a low-grade cancer with an indolent course, but high-grade tumors can rarely show aggressive behavior, especially those with dural extension. ONB is typically treated with surgery followed by radiotherapy while systemic treatment is usually palliative and limited to patients with metastatic disease. There is no standard systemic treatment for R/M ONB. However, similar to other small-cell malignancies, the most commonly used chemotherapy regimen is cisplatin plus etoposide. Limited data have been reported on targeted therapies, while there are no data on immunotherapy which is an off-label therapy for this rare cancer. Hoshi et al. found promising efficacy results for anti-PD1 therapy with nivolumab or pembrolizumab, opening up a possible alternative to chemotherapy in ONB, for which systemic treatment remains limited and not standardized. The lack of apparent association with PD-L1 expression highlights the need to identify predictive biomarkers even in this rare cancer.

Chen M et al. contributed to this Research Topic by reporting the molecular characterization of nuclear protein in testis (NUT) carcinoma in two patients and reviewing the literature on the pathologic and molecular features. NUT carcinoma is one of the rarest and most aggressive sinonasal cancers with a very poor prognosis and a median overall survival of less than 12 months. The authors found that NUT carcinoma typically presents with undifferentiated or poorly differentiated small cells, sometimes with evidence of focal abrupt keratinization, and with variable expression of p40 and CK protein. All cases with available data stained positive for NUTM1 protein and showed heterogeneous genotypes including different NUTM1 fusion partners, with a, low tumor mutational burden and stable microsatellites. There is currently no standard treatment for NUT carcinoma. The mainstay is a multimodal approach including surgery, radiotherapy and possibly systemic therapy. Targeted therapy with histone deacetylase inhibitors (HDACi) and BET inhibitors are under investigation. The molecular characterization of NUT carcinoma may provide new opportunities to test new drugs in this poor-prognosis patient population.

Three interesting case reports describe the management of different sinonasal cancer subtypes. In the first case reported by Chen F et al., the authors describe the case of a patient with sinonasal squamous cell carcinoma who was treated with 2 cycles of neoadjuvant docetaxel and cisplatin chemotherapy plus the anti-PD-1 agent tislelizumab. To date, there is no established role for neoadjuvant treatment in resectable sinonasal squamous cell carcinoma, and immunotherapy-based treatment is not approved for sinonasal cancers. However, preoperative systemic therapy may be of potential benefit in reducing tumor size and achieving adequate surgical margins, which has been shown to impact survival in large retrospective series. The complete pathologic response observed in the clinical case provides a signal to further investigate the potential benefit of early systemic therapy in selected cases. This strategy may be particularly useful in patients where invasion of critical structures could compromise both the adequacy of surgical margins and organ function.

Another case report by Cubides-Cordoba et al., focuses on human papillomavirus (HPV)-related multiphenotypic sinonasal carcinoma (HMSC), a new entity recently recognized in the latest WHO classification and provisionally included in the previous edition as “HPV-related carcinoma with adenoid cystic-like features”. It is characterized by myoepithelial differentiation, squamous atypia, and the presence of high-risk HPV (usually type 33, followed by type 35) with strong and diffuse positivity for p16. The authors present a clinical case of a patient with HPV 35-associated HMSC, in whom whole exome sequencing (WES) revealed some potential targets for personalized treatment.

The final case report by Quan et al., describes an unusual and rare case of coexistence of small cell lung cancer (SCLC) and primary sinonasal small cell carcinoma. Sinonasal small cell neuroendocrine carcinoma is an aggressive and extremely rare cancer for which there is no standard treatment. The authors highlight the need for an accurate immunohistochemical and genomic characterization of this rare cancer, as well as the potential benefit of multimodal treatment combining platinum-based chemotherapy, immunotherapy and local radiotherapy.

## Concluding remarks

Sinonasal cancers and treatment strategies for these cancers are poorly understood. Molecular and genetic information will help identify new entities and subgroups of tumors with different prognosis, offering the possibility of developing new treatment strategies and personalized treatments in the future. We hope that readers of this Research Topic will find it a useful reference point for directing additional research attention to this rare and heterogeneous group of cancers.
